# Connecting biology, optics and nanomechanical properties in micro-wasps

**DOI:** 10.1038/s41598-020-58301-2

**Published:** 2020-01-29

**Authors:** Rebeca Mora-Castro, Marcela Hernández-Jiménez, Giovanni Sáenz-Arce, Juan Porras-Peñaranda, Paul Hanson-Snortum, Esteban Avendaño-Soto

**Affiliations:** 1 Centro de Investigación en Biología Celular y Molecular, Universidad de Costa Rica, 11501 San Pedro de Montes de Oca, Costa Rica; 2 Escuela de Biología, Universidad de Costa Rica, 11501 San Pedro de Montes de Oca, Costa Rica; 3 Centro de Investigación en Ciencia e Ingeniería de Materiales and Escuela de Física, Universidad de Costa Rica, 11501 San Pedro de Montes de Oca, Costa Rica; 4 Departamento de Física, Universidad Nacional, 86-3000 Heredia, Costa Rica; 5 Sección de Patología, Hospital San Juan de Dios, San José, Costa Rica

**Keywords:** Nanoscale biophysics, Atomic force microscopy, Cryoelectron microscopy, Biomaterials

## Abstract

Coloration in insects provides a fruitful opportunity for interdisciplinary research involving both physics and biology, and for a better understanding of the design principles of biological structures. In this research we used nanometric and micrometric analyses to investigate the morphological and mechanical properties of the black-orange-black (BOB) color pattern in scelionid wasps, which has never been studied. The primary objective of the present investigation was to explore the structural and mechanical differences in the mesoscutum of four species: *Baryconus* with an orange mesosoma (i.e. BOB pattern), all black *Baryconus*, *Scelio* with an orange mesosoma (i.e. BOB pattern), and all black *Scelio*. The most outstanding findings include the absence of multilayer structures that generate structural color, a pigment concentrated in the upper surface of the epicuticle, and surprising differences between the four species. Three of the four species showed an accordion-like structure in the furrow (notaulus), whereas the adjacent mesoscutum was different in each species. Moreover, the normalized color component spectra for blue, green and red colors of the black mesoscutum of each genus showed the same spectral dependence while the orange color manifested small changes in the dominant wavelength, resulting in slightly different orange tones.

## Introduction

Biological systems on virtually any scale provide real opportunities for advancing our understanding of both the physics and biology of organisms, serving as models and trusted sources of inspiration. Within this wide range of biological examples, insects stand out. They are some of the oldest animals on the planet, having survived a diverse range of environmental conditions. Among the insect features allowing for fruitful interdisciplinary research are their colors.

Coloration in insects can be generated by numerous forms of surface and epidermal structures (structural colors), by the deposition of different chemical pigments in the outer body layers that selectively reflect, absorb or scatter specific wavelengths of light or by a combination of both mechanisms. Pigments are responsible for most of the yellow, orange, red, and brown-black colors observed in insects while most green or blue colors result from nanostructured features that reflect these colors^1^. To accomplish these intricate designs, cuticle must be an adaptable and versatile material, being light, tough, rigid, flexible, elastic, rubbery, solid or porous, isotropic or anisotropic, as the needs required^2^.

**Figure 1:**
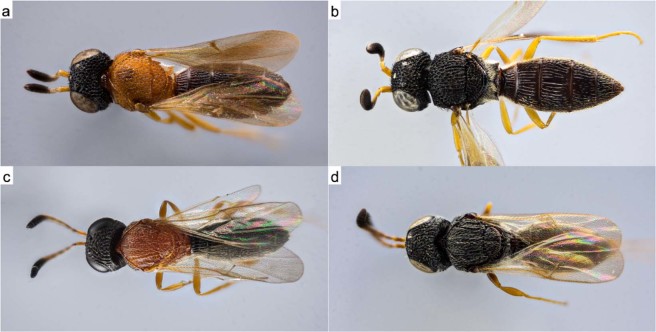
Macrophotography of dorsal color patterns of two scelionid genera. Macrophotography of dorsal color patterns of two scelionid genera obtained with a Reflex Camera 850, 20X microscope lenses and results of focus stacking of 180 captures. Genus *Scelio* (**a**) orange morph (SO), (**b**) black morph (SB) and genus *Baryconus* (**c**) orange morph (BO), (**d**) black morph (BB).

Compared with Lepidoptera and Coleoptera, there are relatively few studies of morphological and mechanical properties of cuticle color in Hymenoptera, and they are mostly restricted to Vespinae^3–6^. However, it has long been recognized that many hymenopterans, especially scelionids, show a recurring color pattern of black head, orange mesosoma, and black metasoma (BOB) (Fig. 1)^7^ and appears to occur in 90% of the currently known species of *Chromoteleia*, 70% of *Acanthoscelio* and *Triteleia*, 50% of *Baryconus*, 40% of *Pseudoheptascelio*, 30% of *Opisthacantha*, *Scelio* and *Sceliomorpha*, and 15% of *Macroteleia*^8^. However, no final conclusions about the function and mechanism of this color pattern have been drawn so far. It seems unlikely that this color is used in inter-sexual communication since both sexes usually have the same color, and most of the intraspecific variation reported included color variation within the same sex^7^. The BOB color pattern has been documented to occur in at least 23 families of Hymenoptera; while scelionid larvae are parasitoids of insect eggs, this color pattern occurs even in some sawflies, which have phytophagous larvae. Hymenopterans with the BOB color pattern are mostly small in size (between 3 mm and 10 mm) and are especially common in the Neotropics where they occur primarily at lower elevations; as far as known they are diurnal^7,9^.

Obtaining fresh specimens of scelionid wasps with BOB coloration for chemical analysis is extremely difficult since the hosts (eggs of crickets and related insects) are nearly impossible to find in the field and no laboratory cultures are currently available. At the present time fresh specimens can only be obtained by sweeping the vegetation with an insect net, which requires many hours to obtain just a few specimens. For this reason a previous study used microspectrophotometry to study the spectral properties of the orange and black colors, and the results suggested that there is a common compound for the pigments^10^. The primary objective of the present investigation was to further explore the physical basis of these colors; more specifically, our aim was to investigate the structural and mechanical differences between orange and black mesosoma in each of two genera, *Scelio* and *Baryconus*. In addition, an understanding of the nanomechanical properties of the cuticle of these micro-wasps may provide valuable background information and suggest directions for future research.

## Results

### Scanning electron microscopy, cryofracture and eosin hematoxylin

**Figure 2:**
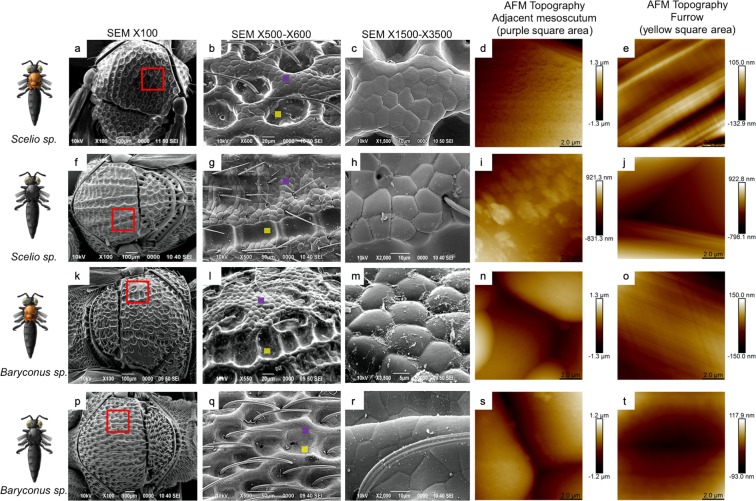
SEM and AFM topography of *Scelio* and *Baryconus*. SEM and AFM topography for the mesosoma of: *Scelio* orange (SO) (**a–e**), *Scelio* black (SB) (**f–j**), *Baryconus* orange (BO) (**k–o**) and *Baryconus* black (BB) (**p–t**). The red box points out the area studied at different scales in SEM and the small purple and yellow filled squares indicate the precise spot, from the red marked area, analyzed with AFM.

Different magnifications of the SEM images show the main features of the morphology of the specimens coated with a thin layer of gold to avoid charging. The cuticle of all specimens of both morphs is quite intricate and sclerotized (Fig. 2a,f,k,p) as reported previously^9^. In the small fragment that was analyzed, one main structure stands out in both genera and both morphs (black and orange): pentagonal and/or hexagonal shaped structures. Observing in more detail shows either rosette-shaped structures with six pentagonal and/or hexagonal projections as in *Scelio* (Fig. 2a–c,f–h) or a repeatable and symmetrical pentagonal and/or hexagonal covering along the entire surface as in *Baryconus* (Fig. 2k–m,p–r). Several of these rosettes or repeatable structures showed a central pore, some of them featuring setae. Another evident structure in SB and BO is a furrow, known in the entomological literature as the notaulus (Fig. 2g,l). However, the cuticle as a whole in BB seems to be less flat exhibiting an undulating surface (Fig. 2r).

**Figure 3:**
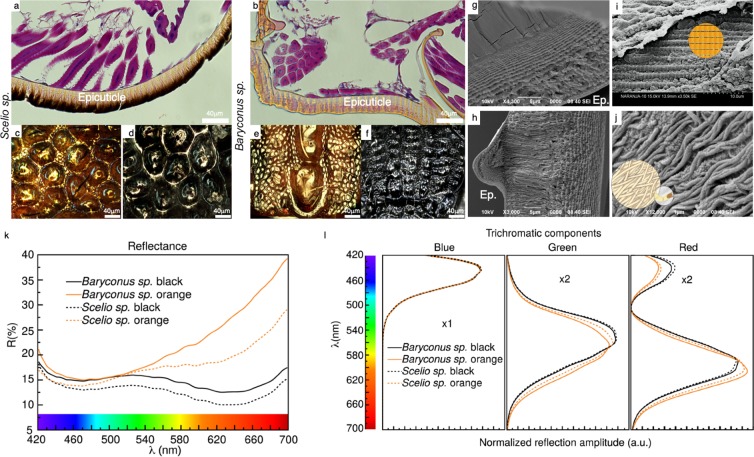
Optical image of *Scelio* and *Baryconus*. As a result of eosin hematoxylin technique, the mesosoma of: *Scelio* black (**a**), orange (**c**), black (**d**) and *Baryconus* orange (**b**), orange (**e)**, black (f) is observed. Ep: epicuticle. Cryofractures of *Scelio* black (**g**) and *Baryconus* orange (**h**), highlighting details: *Scelio* showing lamellae (**i**) and *Baryconus* showing fibers (**j**). Reflectance spectra for black and orange mesosoma are shown in inset (**k**), while inset (**l**) shows the corresponding spectral components.

As mentioned before, the cuticle of these specimens is far from being flat and simple, even the thickness varies in the different parts of the mesoscutum (part of interest). It presents many irregularities and the subsidences, hooks, peaks and curvatures are manifested in the internal architecture of all layers of the cuticle (Fig. 3a–f). Cryofractures of the dorsal thorax (mesonotum) cuticle revealed a total thickness of about ≈28 *μ*m–50 *μ*m (BO) (Fig. 3h) and ≈15 *μ*m–40 *μ*m (SB) (Fig. 3g). The cuticle of BO is composed of approximately 15–19 lamellae (Fig. 3h) whose thickness diminishes as one proceeds from the outer covering inwards while the cuticle of SB consists of 17–23 lamellae (Fig. 3g). The outermost part of the cuticle, the epicuticle, appears as a compact and homogenous layer of ≈0, 5–1 *μ*m in BO and SB. Multilayer structures or other interference structures were not observed in this area, neither in the black or orange morphs. It is important to mention that in this layer clearly deposited black and orange pigments were observed (Fig. 3a,b). Interestingly, in SB a very subtle orange color was also observed embedded in the lower layers of the epicuticle, as if it was embedded below the black pigment in lower amounts (Fig. 3a). With respect to the procuticle there are two conformations clearly present, the lamellae mentioned before (lower layers of the procuticle) and another ordering, just below the epicuticle: in SB it appears to be composed of an intralayer microstructure of irregular compartments (Fig. 3g), differing moderately from what was observed in BO, which appears more like a longitudinal columnar pattern (Fig. 3h). In both genera, in the deepest part of the procuticle, what some authors previously called the endocuticle, a stack-like arrangement of lamellae (Fig. 3i) is observed, layers appearing to be of equal thickness, but the space between the individual layers varies.These layers appeared chitin based and it seems that between those layers a filling, perhaps proteinaceous, could be present. Another noticeable detail is that the layers seem divided by frequent ringed partitions. According to Fig. 3j, which is a magnification of the possible filling material mentioned before, it appears to contain fibers, of possible protein origin, with mesh-like and irregular configurations.

### Reflectance spectra and spectral components

Reflectance spectra for black and orange segments are shown in Fig. 3k. As in cryofracture, the eosin hematoxylin technique revealed features of the internal structure of the cuticle. In those structures, the black pigment seems to have a more compact and localized distribution at the upper edge of the epicuticle (Fig. 3a) than the orange pigment (Fig. 3b). Because of the relatively low reflectance values, it can be inferred that most of the light is dispersed within the cuticular structure, and that the spectral characteristics are generated mainly by the pigment in the epicuticle. Therefore the reflectance spectra correspond primarily to information obtained from epicuticular structures within a thickness of no more than 10 *μ*m.

For short wavelengths, the curves have a similar behavior up to 520 nm approximately. For longer wavelengths, there is a clear difference between orange and black, the two black samples being very similar. In terms of spectral components (Fig. 3l), there is no difference between the blue components even when considering different genera (*Baryconus* vs. *Scelio*) or even different colors (black vs. orange). Differences between curves become appreciable in the green and red components. In such cases orange and black reflectance curves are different, but are the same in terms of genus, i.e., *Scelio* orange (black) has the same spectral behavior as *Baryconus* orange (black). In a prevous work^10^ a statistical analysis showed that these observed differences and similarities are statistically significant even when considering factors such as different specimens and measurement spots. Thus, the normalized color component spectra for the blue, green and red colors, of the black mesoscutum on each genus, show the same spectral dependence. This means that both blacks are the same with the only variation being the hue of the color. Irrespective of the genus or the orange vs black mesoscutum, the blue component had the same spectral dependence, with only differences in the area when not normalized. In the case of the color component for the orange variation in both genera, similar behavior is observed, but small changes in the dominant wavelength are observed, resulting in slightly different orange tones. Further analyses, which also include other genera of scelionid wasps, can be found in a previous publication^10^.

### Atomic force microscopy

**Figure 4:**
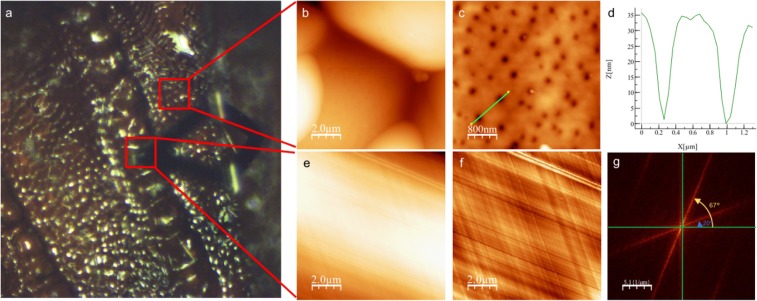
*Baryconus* orange AFM topography. (**a**) Optical image of *Baryconus* orange mesosoma. (**b**) Adjacent mesoscotum, which corresponds mostly to the hexagonal and/or pentagonal structures. (**c**) High resolution of the surface of a granule. (**d**) Profile of two pores. (**e**) Inside the furrow. (**f**) Image **e** with a second order filter where a fibrillar structure is shown. (**g**) Fast Fourier Transform of image **f**.

Figure 2 shows the morphology measured by means of two techniques: scanning electron microscopy (explained above) and atomic force microscopy. For the latter, a peak force method was used in order to generate the image. Two distinct regions and their features were measured, inside the furrow (notaulus) and adjacent mesoscotum, which corresponds mostly to the hexagonal and/or pentagonal structures mentioned before. The AFM images show the same morphological features as in SEM but with better detail of the surface characteristics, because there is no need for a gold coating or exposure of the sample to vacuum as in the SEM preparation. The peak force method allows the tip to be submerged into the water layer covering the sample without affecting the topographic information. The surfaces in general appear to be very smooth in the nanometric scale. The roughness inside the furrow is approximately: 24.5 nm (SO), 23.9 nm (BO), 31.5 nm (SB) and 25.9 nm (BB). For the adjacent mesoscotum, the roughness values are 181 nm (SO), 211 nm (BO), 362 nm (SB) and 323 nm (BB). The hexagonal and/or pentagonal structures in *Scelio* (approx. 12 *μ*m each) are more flat and regular with respect to *Baryconus* (approx.10 *μ*m each), in which those features are more rounded. Figure 4 shows the detail of the *Baryconus* orange morphology. The hexagonal and/or pentagonal features outside the furrows show a surface densely covered with pores, with an outermost diameter of approximately 0.5 *μ*m. It is not possible to quantify the depth of these pores because of the limitation of the aspect ratio of the tip with respect to dimensions of the pores. The roughness between the pores is 1.3 nm. Inside the furrow, a fiber like structure is shown. The Fast Fourier Transform analysis shows a preferential direction in the ordering of the fibers, in which an angle of 47° was measured.

**Figure 5:**
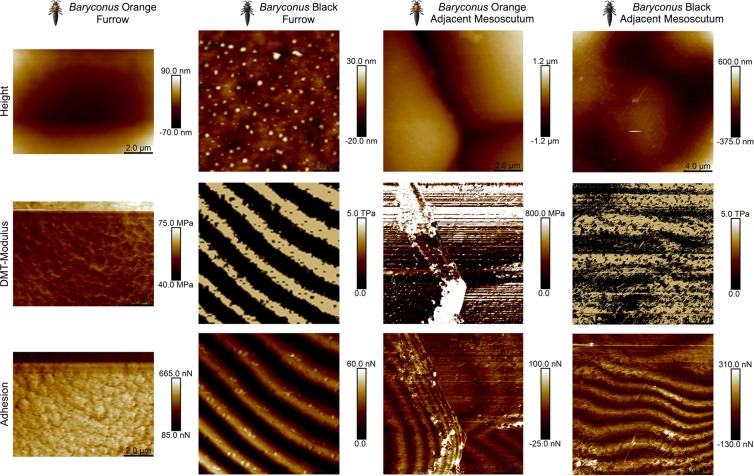
Peak Force AFM images for *Baryconus* orange and black mesosomas. Peak Force AFM images for the *Baryconus* orange and black mesosomas where the rows are ordered from top to the bottom as follows: Height, DMT-modulus and Adhesion. The columns are grouped in pairs as furrow (notaulus) and adjacent mesoscutum for the orange (BO) and black (BB) variations of *Baryconus*.

**Figure 6:**
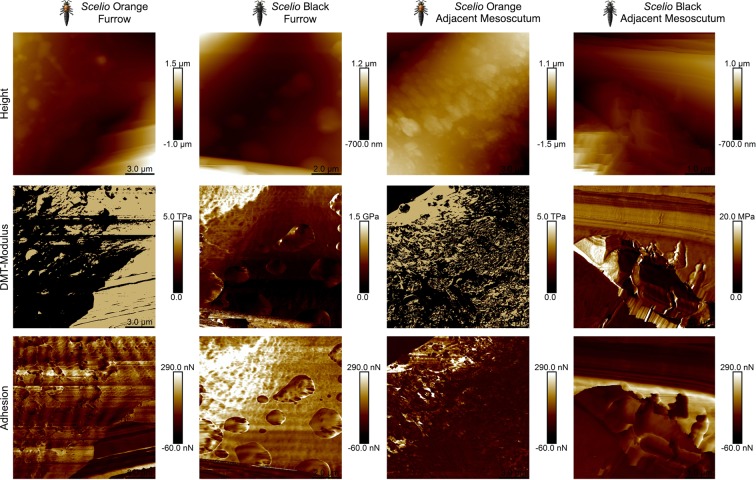
Peak Force AFM images for *Scelio* orange and black mesosomas. Peak Force AFM images for the *Scelio* orange and black mesosomas where the rows are ordered from top to bottom as follows: Height, DMT-modulus and Adhesion. The columns are grouped in pairs as furrow (notaulus) and adjacent mesoscutum for the orange (SO) and black (SB) variations of *Scelio*.

**Figure 7:**
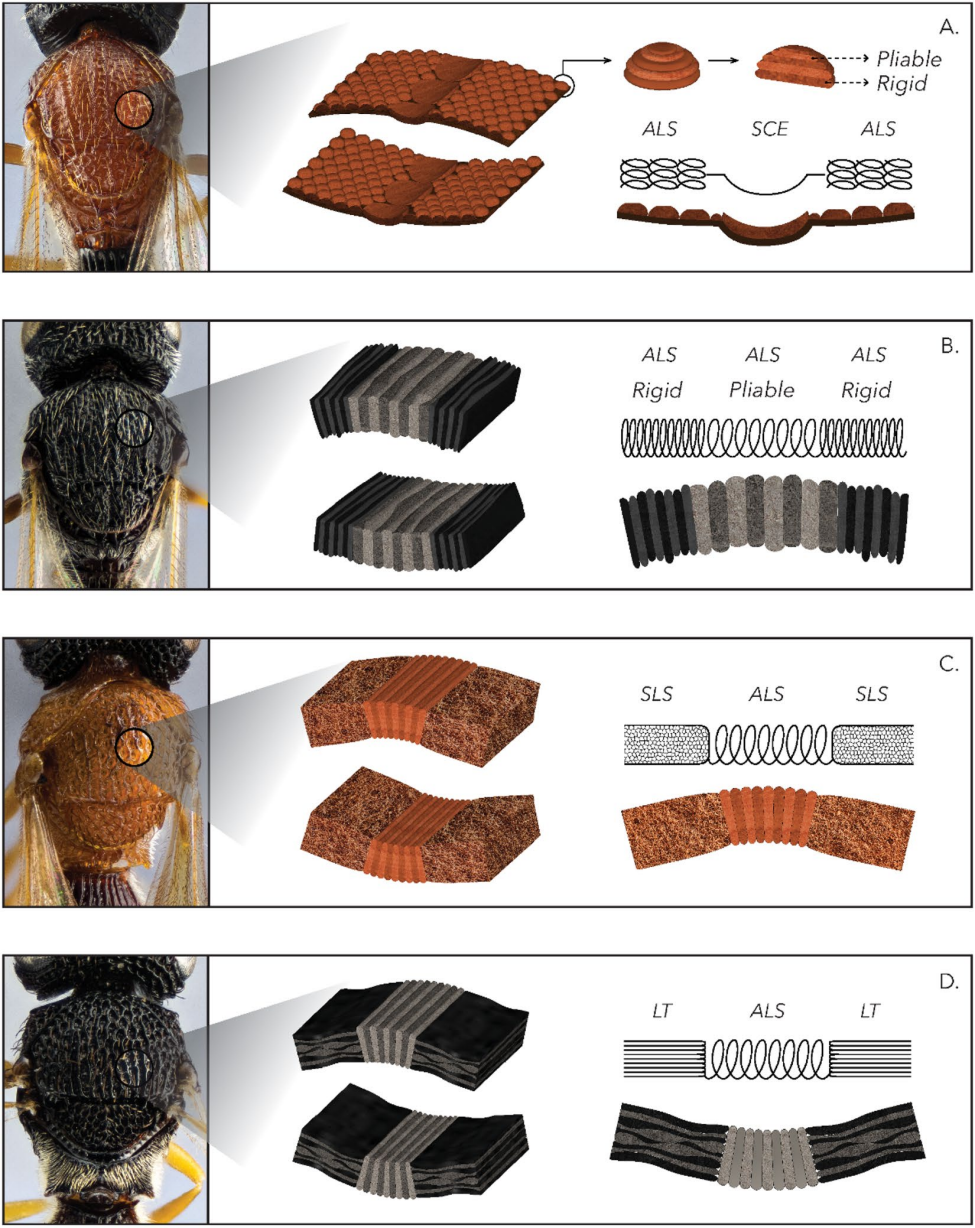
Graphic representation of structures observed by means of AFM. Graphic representation in 3 D modeling of the six major distinct structures that were observed by means of peak force AFM in the orange and black specimens of *Baryconus* (**A,B**) and *Scelio* (**C,D**). In the case of the furrows for the two genera, there are two variations, accordion-like structures (ALS) (**B–D**) or soft connecting structure (SCE) (**A**). For the adjacent mesoscotum there are four variations, granulated stacked accordion within an accordion-like structure (ALS)(**A**), accordion-like structures (ALS) (**B**), sponge-like structures (SLS) (**C**) and lamellae terraces (LT) (**D**).

Figures 5 and 6 show the morphological and nanomechanical images of the BOB pattern for the two genera. The figures include the Height, DMT-modulus and Adhesion information. Figure 7 is a graphical representation in 3 D modeling of the six major structures that were observed by means of peak force AFM in Figs. 5 and 6. In the case of the furrows for the two genera, there are two variations, an accordion-like or soft connecting structures. For the adjacent mesoscutum there are four types: granulated stacked accordions, sponge-like, lamellae or accordion-like structures.

The *Baryconus* orange mesoscutum presents two distinct structures, the granulated agglomerations and the furrow between them. The furrow is a soft connecting structure with a DMT-modulus on the order of 50 MPa. The granulated structure that comprises the mesoscutum has rounded structures (more hexagonal/polygonal-like in SEM) approximately 12 um of diameter and presents a DMT-modulus on average 10 times higher. Each grain shows a layered structure that resembles a laminated or stacked spring. All structures together form a resonant like chamber.

The *Baryconus* black presents two similar structures for the furrow and adjacent mesoscutum, and both structures resemble an accordion. The furrow has intercalated layers of stiff and soft material more or less parallel and regular with respect to each other that lie between the furrow structures with a thinner intercalated layered structure with undulated shapes. The furrow layers are almost double in thickness with respect to that of the adjacent mesoscutum.

The *Scelio* orange has a similar furrow structure as the *Baryconus* black, but the furrow resemble a sponge-like structure, with a composite of soft and hard materials. Finally, the *Scelio* black presents the same accordion-like furrow, but the adjacent mesoscutum structure is composed of large lamellae with intercalated layers of stiff and soft material. There are much larger structures than those found in the *Baryconus* orange furrows.

## Discussion

The cuticular structure of very small insects, such as the micro-wasps examined here, has generally been neglected. Comparing our results with those of a much larger wasp (*Vespa orientalis*)^3,4,11^ some similarities are evident. For example, the cuticle of the specimens studied here exhibit a composite material in which protein-like matrices, lamellae and fibers were observed. Other characteristics also show certain similarities, for example the gradually decreasing thickness of lamellae, similar ranges of thickness of cuticular layers and the presence of pores. Some of the surface characteristics are widespread in insects; for example, pores have been reported in various beetle families (Coccinellidae, Dystiscidae, Lucanidae, Scarabaeidae) but the microstructure and the density of these pores can vary^12,13^. Orange and black colors are generally due to pigments as opposed to interference structures in the cuticle^1^ and the results of the present investigation clearly suggest that these colors in scelionid wasps are not structural in nature. To the best of our knowledge, Mutillidae is the only group of Hymenoptera in which the chemical basis of BOB coloration has been examined, namely orange pheomelanins and black eumelanins^14^. The fact that all blue components of the spectral analysis coincide, while the green and red components differ between black and orange, suggests that there is a shared chemical composition. Thus, information obtained from the reflectance spectra suggests that the coloration is due to pigments that are closely related in terms of chemical composition, as discussed in a previous work^10^, and that such pigments are encapsulated within the epicuticle (Fig. 3a,b).

Nonetheless, optical micrographs of the cuticular surface of the corresponding mesoscutum (Fig. 3c–f), show that the coloration is not necessarily homogeneous. This is especially evident for the orange mesoscutum of both *Scelio* and *Baryconus*, and this effect could also account for the similarities found between orange and black reflectance spectra.

One of our objectives was to ascertain how the properties of black cuticle differ from those of orange cuticle. However, it should be noted that the results from the Peak Force AFM technique are composed of different contributions (structure, topography and chemical composition) and should be interpreted within this holistic context. In order to clarify whether the color has an influence on the nanomechanical properties, the chemical composition of the pigments needs to be determined and then more experiments should be carried out. In fact, when comparing the four possible combinations (orange vs black and *Baryconus* vs *Scelio* the rather surprising result was that each combination is quite different (Fig. 7). The orange mesoscutum in *Baryconus* (Fig. 7A), is made up of granular structures, each rounded structure consisting of alternating pliable and rigid layers, while the furrow (notualus) appears to be a softer connecting structure. The black mesoscutum in *Baryconus* (Fig. 7B) has an accordion-like structure in both the furrow and the adjacent mesoscutum, the latter being more rigid. The furrow has intercalated layers of stiff and soft material, more or less parallel to each other, and almost double in thickness with respect to the adjacent mesoscutum. The orange mesoscutum of *Scelio* (Fig. 7C), has a sponge-like structure, with a composite of soft and hard materials; the furrow has an accordion-like structure. Finally, the black mesoscutum of *Scelio* (Fig. 7D), consists of horizonal layers of lamellae, with intercalated layers of stiff and soft material; the furrow has an accordion-like structure similar to the previous two combinations.

Presumably all four combinations discussed above permit the flexibility required as the mesothorax becomes deformed during flight (due to the action of the indirect flight muscles), but further research is required to better understand the notable differences observed in Fig. 7. Within the mesoscutal furrow of *Baryconus* a fiber-like structure was found with a preferential direction in the ordering of the fibers showing an angle of 47°, Fig. 4f,g. The value of the angle between fibers has been reported to indicate higher ductility of the material^15^ meaning that this characteristic allows greater deformation with respect to other structures^15,16^.

Biological studies are required to determine whether the BOB pattern serves as a warning (aposematic) coloration for potential predators and whether the black colors play a role in thermoregulation. Addressing these questions would be greatly facilitated by being able to rear scelionids in the laboratory, which will require rearing of their hosts, namely the eggs of crickets or katydids. Lab rearing of scelionids would also allow studies of cuticle development, for example cuticle from adults recently emerged from the pupa versus older wasps. Moreover, phylogenetic studies of scelionids as a whole, and of individual genera, would allow us to address the question of how many times this coloration has evolved.

Many additional questions remain regarding the BOB color pattern that is so prevalent among scelionids. How do the contrasting black and orange colors interact in the visual system of potential predators or other receivers of these visual signals? How do the superficial and internal structures reported here interact? What are the pigments producing the orange and black, to what degree do pigments vary among scelionids (and other wasps showing the BOB pattern), and to what extent do the structures in which the pigments are deposited affect the color as viewed by signal receivers. A few species show color polymorphism^7^, a phenomenom that poses numerous additional questions. We obviously have much more to learn from these micro-wasps.

## Methods

### Specimens and samplig

Live specimens of Scelionidae are extremely difficult to find in the field because of their small size and concealed nature of their hosts (insect eggs). In order to obtain fresh specimens with internal and external structures in good condition, our field collecting focused on two common scelionid genera, *Scelio* and *Baryconus*, which were captured with fine entomological nets. All experimental protocols were carried out in accordance with the institutions’ guidelines and regulations. The taxonomic classification of the specimens was carried out in the laboratory on the same day of the collection. Collecting was done in El Rodeo, which is part of the Mora County (Municipality), located 30 Km southwest of San Jose, Costa Rica, within a natural reserve of a secondary forest and a remnant of primary forest (200 ha) in the Central Valley of Costa Rica. This site provided the greatest abundance (1 to 3 specimens collected per hour) and diversity of the group (eight Scelionidae genera were found with BOB color pattern), which is why it was chosen as the definitive field site for this study. The specimens were freeze-killed and immediately analyzed with the techniques mentioned below.

Five complementary techniques were used in this study and were intended to provide the data and information needed to generate a design principle that may elucidate the nature of the orange and black colors in Scelionidae: micro spectrophotometry, eosin hematoxylin, scanning electron microscopy, cryofracture and atomic force microscopy. A minimum of three specimens of each genus were analyzed with each technique. The techniques analyzed fragments of no more than 3 mm extracted with entomological microneedles from the mesosoma, directly in the mesoscutum of the specimens.

### Microspectrophotometry and spectral components

In order to study the spectral characteristics of the cuticle of these insects, we used a microspectrophotometer (508PV, CRAIC), in the visible - near infrared portion of the electromagnetic spectrum, coupled to a trinocular microscope (Eclipse LV200ND, Nikon) in episcopic illumination mode. Reflectance spectra were normalized using a Spectralon white reference (see Table 1). Rather than color coordinates in a predetermined color space, we used the CIE 1931 color-matching functions (CMFs) to obtain three components for each reflectance spectrum in a RGB manner. Further description of this analysis can be found in ref. ^10^. Components were defined in the following way:

1 $$R(\lambda )=\frac{Y(\lambda )\bar{x}(\lambda )}{\int Y(\lambda )\bar{x}(\lambda )d\lambda },G(\lambda )=\frac{Y(\lambda )\bar{y}(\lambda )}{\int Y(\lambda )\bar{y}(\lambda )d\lambda },\,{\rm{and}}\,B(\lambda )=\frac{Y(\lambda )\bar{z}(\lambda )}{\int Y(\lambda )\bar{z}(\lambda )d\lambda },$$

where *Y*(*λ*) is the reflectance spectrum as a function of wavelength *λ* and $\bar{x}(\lambda )$, $\bar{y}(\lambda )$ $\bar{z}(\lambda )$ are CMFs regarding the CIE 1931 standard (2°) colorimetric observer.

**Table 1:** Information about the analysis of color data.

Microspectrophotometry technical details	Information
Light source	12 V–100 W halogen lamp
Magnification	5X, CFI60 2 TU Plan Fluor BD,
	N.A 0.15, WD 18.0 mm, (Nikon,Tokyo,Japan)
White reference	Spectralon Diffuse Reflectance Standard
	USRS-00-010 (Labsphere, North Sutton,NH, USA)
Dark reference	Internal attenuators
Software for spectral capture	Lambda Fire (CRAIC,Los Angeles,USA)
Integration time	150 ms
Number of spectra averaged	100

Even though the CMFs are related to human visual sensibility, the aim of studying the reflectance spectra by components is to access information about differences in the chemical composition of the pigments involved in coloration and that might be inferred from the spectral characteristics of the reflectance. As discussed in a previous work by our group^10^, the study of optical properties is related to the interaction of the electronic structure of the material with electromagnetic radiation, and therefore is an indirect chemical characterization. Morever, this procedure can be eventually adapted to study the relevance of spectral characteristics when considering visual sensibilities of a given insect.

### Eosin hematoxylin

Each of the specimens was fixed in 10% formalin, where they were preserved until they were processed. Subsequently, a post fixation in formaldehyde, acetic acid, ethanol solution was carried out for two hours. Dehydration was carried out in alcohols with increasing concentration (70%, 95%, 100%). Clarification was implemented with xylol and later the sample was impregnated with paraffin. Once the specimens were processed, they were included in paraffin blocks. Continuous cuts were made at 4 microns in thickness and 3 or 4 cuts were placed per slide, using the entire mesoscutum. Hematoxylin and eosin (VENTANA HE 600 system) stains were performed on all sections. The final cuts were observed in an Olympus bx51 microscope and the photographs obtained with an Olympus Dp72 camera.

### Scanning electron microscopy

The samples were analyzed using a SEM JSM-5900 LV (JEOL, Tokyo, Japan), voltage 10–20 kV, pressure of 1 × 10^−4^ Pa. The biological samples were fixed with glutaraldehyde 2%, paraformaldehyde 2% in phosphate buffer (PB) 0.05 M pH 7.2 at 4 °C for 48 hours, washed in PB 0.05 M pH 7.2, post-fixed in OsO4 2% in PB 0.05 M pH 7.2 and dehydrated in a serial gradient of acetone solutions, being finally dried with carbon dioxide in a critical point dryer LEICA EM CPD 300. Lastly, the samples were coated with a 20 nm gold layer in a sputter coater EMS 550X. The images were analyzed at 200 to 2500X magnifications. A carbon tape or freshly cleaved mica on top of a SEM sample holder was used to support the samples.

### Atomic force microscopy

Atomic force microscopy images and nanomechanical data were collected using a Multimode 8 microscope (Bruker, USA) operating in PeakForce Tapping mode. The specimens were imaged in room condition using ScanAsyst-Air probes (Bruker) (nominal length 115 *μ*m, tip radius 2 nm, spring constant 0.4 N m^−1^) for topography and RTESP-300 probes (Brukes) (nominal length 125 *μ*m, tip radius 12 nm, spring constant 40 N m^−1^). Prior to imaging, the probes were calibrated according to the manufacturer’s protocol using the Bruker calibration kit. Images were collected in height sensor, peak force error, Young modulus (DMT model) and adhesion channels. The raw AFM data obtained were processed using Nanoscope Analysis v.1.7. software (Bruker).

## Data Availability

The datasets generated during and/or analyzed during the current study are available from the corresponding author on reasonable request.
